# Acute Effects of Intermittent High-Intensity Exercise on Cardiac Autonomic Regulation in Male Non-Elite Badminton Players: A Multi-Point Time Series Analysis

**DOI:** 10.3390/healthcare14070864

**Published:** 2026-03-27

**Authors:** Heping Huang, Hongfei Jiang, Huiming Huang, Shenguang Li, Su Liu

**Affiliations:** 1 Institute of Physical Education and Sports, Huzhou Normal University, Huzhou 313000, China; 02773@zjhu.edu.cn (H.H.); zgyd1012@163.com (H.J.); 02418@zjhu.edu.cn (S.L.); 2 College of Physical Education and Health, China Pharmaceutical University, Nanjing 210009, China; huanghuiming@cpu.edu.cn

**Keywords:** heart rate variability, autonomic nervous system, intermittent exercise, exercise-induced fatigue, non-elite players

## Abstract

**Objective**: This study aimed to investigate the acute effects of intermittent high-intensity badminton court exercise on cardiac autonomic modulation in male non-elite badminton players. **Methods**: This study employed a single-arm, repeated-measures experimental design, recruiting 25 healthy male collegiate badminton players. Participants completed five sets of high-intensity intermittent court tests until exhaustion, followed by calculation of stress index (SI), time-domain (RMSSD and SDNN), and frequency-domain (LF, HF, and LF/HF ratio) parameters at rest using a certified heart rate variability (HRV) analyzer. Repeated-measures ANOVA and effect size (partial η^2^ and Hedges’ g) were used to assess changes and recovery trends of HRV parameters across time points: pre-test, immediate, 15 min, 24 h, and 48 h post-exercise. **Results**: (1) Stress index: The overall temporal trend showed statistical significance (*p* < 0.001, partial η^2^ = 0.236, large effect size). Compared to pre-test, immediate and 15 min post-exercise increases were 8.24 (95% CI: 0.63–15.85) and 9.84 (95% CI: 3.07–16.61) respectively, with Hedges’ g values of 0.77 and 0.99 (*p* < 0.001, large effect sizes). Values returned to pre-test levels at 24 and 48 h with no significant differences (*p* > 0.05). (2) Time-domain parameters: The overall temporal trend was statistically significant (*p* < 0.001, partial η^2^ = 0.553 for RMSSD and 0.586 for SDNN, both large effect sizes). Immediate post-exercise decreases in RMSSD and SDNN were 35.44 (95% CI: 21.95, 48.93) and 48.44 (95% CI: 32.49, 64.38) respectively, with Hedges’ g values of 2.31 and 2.78 (*p* < 0.001, large effect sizes). At 15 min, decreases were 31.64 (17.85, 45.42) and 41.48 (26.23, 56.72) respectively, with Hedges’ g values of 1.99 and 2.25 (*p* < 0.001, large effect sizes). Values returned to pre-test levels at 24 and 48 h with no significant differences (*p* > 0.05). (3) Frequency-domain parameters: Compared to pre-test, differences in LF, HF, and LF/HF were not statistically significant at any time point (all *p* > 0.05). **Conclusions**: Following high-intensity exercise leading to peripheral fatigue, cardiac autonomic function demonstrates a “suppression–recovery” dynamic pattern: cardiac stress levels increase significantly within 15 min post-exercise, with decreased overall HRV regulatory capacity and strong inhibition of parasympathetic activity; HRV status may return to baseline levels after 24 h. However, the frequency-domain indices of HRV showed no significant changes in response to the acute effects of high-intensity exercise.

## 1. Introduction

Badminton is a high-intensity intermittent racket sport characterized by alternating explosive movements with brief recovery periods, including sprints, jumps, and rapid direction changes [1]. The physiological load generated by such sports imposes significant stress on the cardiovascular and autonomic nervous systems, as evidenced by the activation of anaerobic glycolysis and rapid shifts in energy metabolism [2]. Although the physiological response characteristics of elite badminton athletes have been relatively well studied [3], the acute cardiac autonomic regulatory mechanisms in non-elite or amateur players post-exercise remain to be further explored.

Heart rate variability (HRV) is a validated non-invasive indicator that reflects the dynamic synergistic regulation of cardiac rhythm by the sympathetic and parasympathetic nervous systems [4]; acute exercise temporarily alters this regulatory balance [5]. Among time-domain parameters, the root mean square of successive differences between normal heartbeats (RMSSD) and the standard deviation of all normal heartbeat intervals (SDNN) are sensitive indicators reflecting parasympathetic activity levels and overall autonomic regulatory function, respectively [6]. Frequency-domain parameters include high-frequency (HF), low-frequency (LF), and ratio (LF/HF), which further characterize sympathetic–vagal balance and are widely used to assess autonomic responses to physical stress [6]. However, LF/HF has traditionally been used to evaluate sympathovagal balance and the autonomic nervous response to physical stress, although its validity as a marker of sympathetic–parasympathetic activity remains controversial [7]. The prevailing view on post-exercise changes in cardiac autonomic function is that the immediate post-exercise reduction in HRV is a direct and primary manifestation of cardiac vagal tone inhibition [8,9,10,11]. However, reports on the sensitivity of HRV time-domain and frequency-domain analyses in capturing cardiac autonomic function changes following high-intensity exercise are inconsistent. Two studies reported significant reductions in both time-domain and frequency-domain HRV parameters immediately after acute exercise compared with pre-exercise levels [9,10], while two other studies indicated that frequency-domain parameters were unaffected following moderate-intensity continuous training or high-intensity interval exercise [12,13]. Additionally, research has shown that HRV responses to high-intensity exercise differ between populations (e.g., elite vs. recreational athletes) [14,15]. Recent consensus statements underscore that this variability arises from the context-dependent nature of autonomic recovery, influenced by exercise modality, measurement standardization, and individual autonomic responsiveness, rather than inherent ambiguity in physiological mechanisms [16,17]. For instance, compared with elite athletes, recreational players exhibit distinct autonomic recovery dynamics, but their HRV responses following sport-specific exercise loads lack in-depth investigation, particularly regarding HRV recovery patterns in amateur players [18,19].

This study aimed to investigate the acute HRV response characteristics of male recreational badminton players following a sport-specific high-intensity intermittent court test (FT) leading to peripheral fatigue. This study investigated sport-specific loading protocols and analyzed post-exercise HRV recovery at multiple time points in non-elite athletes to elucidate the dynamic changes and recovery trajectories of the autonomic nervous system. The findings will provide evidence for developing personalized training strategies for recreational players, contributing to optimized training load management and monitoring of overtraining risks.

## 2. Materials and Methods

### 2.1. Study Design and Ethical Approval

This study employed a single-arm, repeated-measures experimental design with multiple time points and was conducted in accordance with the Transparent Reporting of Evaluations with Nonrandomized Designs (TREND) guidelines. The study was conducted in accordance with the Declaration of Helsinki and was approved by the Gannan Normal University Medical Ethics Committee (protocol code 2017001, approval date: 11 October 2017).

### 2.2. Participants

Non-elite male collegiate badminton players were recruited through the university’s WeChat platform. Testing was conducted at the laboratory of the Faculty of Sport Science, Gannan Normal University, China, in 2018. Subjects for inclusion were selected by badminton coaches who conducted pre-activity screening and a detailed physical fitness test. In addition, subjects completed a health questionnaire and a physical activity questionnaire.

Inclusion criteria included the following: healthy male participants aged 18 to 25 years who had been screened using the pre-activity screening to confirm a healthy status. Subjects were excluded if they had one of the following conditions: joint or muscle dysfunction, including severe back, neck, knee, and ankle pain; history of having heart disease; serious comorbidity, including cardiovascular or pulmonary disease, coronary artery disease, renal disease and diabetes; injury during the last 6 months; and having hormone therapy at present and/or having a history of hormone therapy. All subjects gave informed consent to participate in this study.

### 2.3. Experimental Protocol

**Figure 1:**
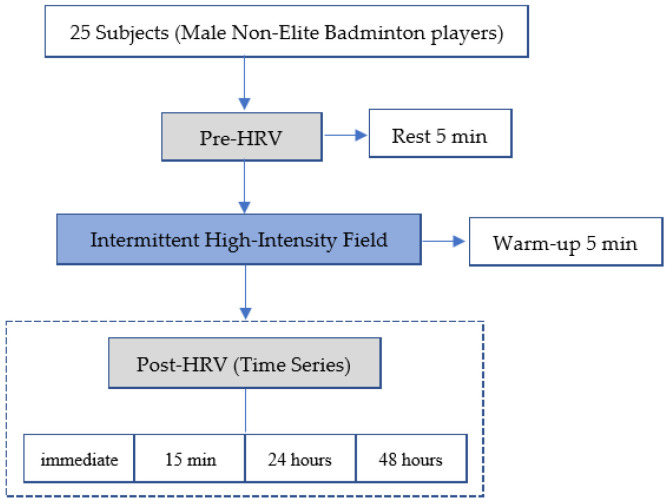
The flowchart shows the protocol of this study.

A time series, single-arm, repeated-measures experiment was conducted with 25 male non-elite badminton players. All participants were instructed to abstain from alcohol and other vigorous exercise for 3 days prior to the experiment until 48 h after the exercise load, and to maintain normal sleep patterns without staying up late. Hydration was provided after the exercise test, but there was no specific requirement to record fluid intake. Following baseline HRV pre-test measurements, subjects performed an intermittent high-intensity field test, after which HRV was repeatedly measured immediately, 15 min, 24 h, and 48 h post-exercise to evaluate the effects of high-intensity exercise-induced peripheral fatigue and its temporal effects on cardiac autonomic function in non-elite players. A flowchart of the subject’s participation and the experimental protocol is shown in Figure 1.

#### Intermittent High-Intensity Field Test

**Figure 2:**
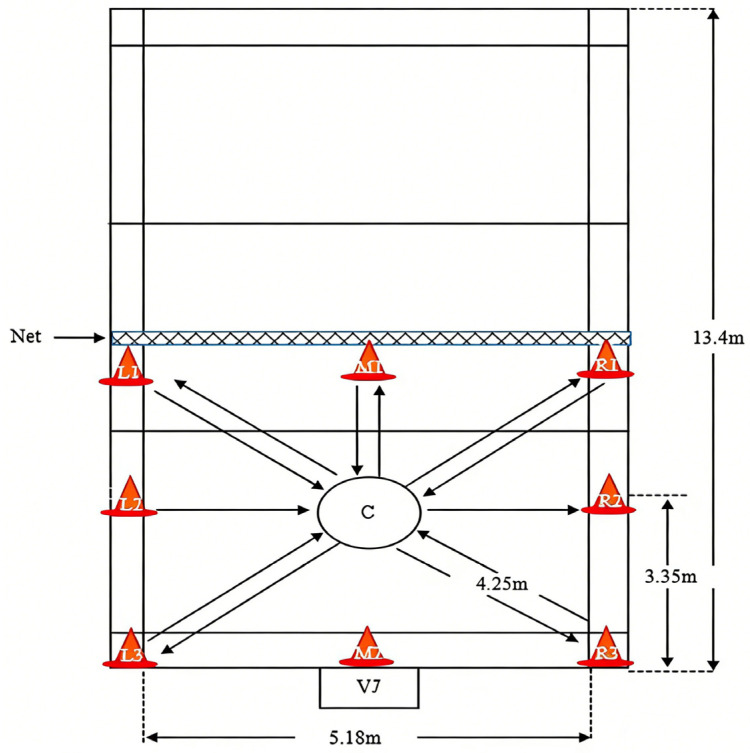
The modified badminton field test with the 8 touch points. VJ: Stands for Vertical Jump; C: Stands for Center; L1/L2/L3 Stands for Left 1/Left 2/Left 3; R1/R2/R3 Stands for Right 1/Right 2/Right 3; M1/M2 Stands for Middle 1/Middle 2.2.3.2. Heart Rate Variability Recording and Analysis.

The intermittent high-intensity field test protocol was designed to simulate the intermittent, high-intensity movement patterns characteristic of badminton match play and has been shown to elicit significant cardiometabolic stress in recreational athletes [20]. The badminton footwork auxiliary trainer with eight flashing lights on a green board was placed on the net in front of the subject. Each light blinked to guide the subject in the direction to run to touch the eight positions/corners on the court. The eight light bulbs corresponded to the forecourt, midcourt, and backcourt. A rear post with a scale in centimeters was measured for the vertical jump at the end of the center point of the badminton court. Subjects were instructed to run and touch each of the eight lamps as quickly as possible, after which they ran back to the rear post to perform the vertical jump. The subjects continued to perform the testing until they achieved one of the following criteria, which were considered as having fatigue: ① cannot run or stopping by participants themselves or ② HR of 92% of HRmax (calculated using the formula: 220 − 0.78 × age) [21,22], or ③ RPE ≥ 18 score [23]. The modified badminton field test with the eight touch points is shown in Figure 2.

Continuous electrocardiogram (ECG) recordings were obtained using a validated HRV analyzer (uBioMaca, Seoul, Republic of Korea) with a sampling frequency of 1 kHz. Participants were seated in a quiet, temperature-controlled laboratory (22 ± 1 °C, 45–55% relative humidity) and underwent a 5 min stabilization period prior to baseline HRV recording. The baseline HRV was then recorded for 5 min in a seated position. The measurements at 24 and 48 h post-exercise were scheduled at the same time points and under the same environmental conditions. HRV parameters were analyzed in both the time and frequency domains using Kubios HRV Premium software (version 3.5). The stress index (SI) was calculated to assess sympathetic tone and reactivity based on heart rate variability [24]. The SI formula is SI = (AMo) × 100/(2Mo × MxDMn), where Mo is the mode, the most common RR interval during a study period; (AMo) × 100 is the % of the number of mode values compared to all values; and MxDMn is the difference between the maximum and the minimum values of the RR intervals. A description of the HRV indicators is shown in Table 1 [25,26].

### 2.4. Statistical Analysis

Data normality was assessed using the Shapiro–Wilk test. Sphericity assumptions were verified using Mauchly’s test; when violated, Greenhouse–Geisser corrections were applied. One-way repeated-measures analysis of variance (ANOVA) with time factors (pre-test, immediately post-exercise, 15 min, 24 h, and 48 h), followed by Bonferroni post hoc tests, was employed to analyze HRV and HR data. For the repeated-measures ANOVA, effect sizes were reported as partial eta-squared (η^2^*p*) and interpreted as follows: small effect (η^2^*p* ≥ 0.01), medium effect (η^2^*p* ≥ 0.06), and large effect (η^2^*p* ≥ 0.14). For pairwise comparisons, we used Hedges’ g as the effect size due to the small sample size and interpreted it as follows: small effect (g ≥ 0.2), medium effect (g ≥ 0.5), and large effect (g ≥ 0.8). The statistical significance level was set at *p* < 0.05. All analyses were performed using SPSS version 26.0 (IBM Corp., Armonk, NY, USA).

We used G*Power 3.1 software, selecting a repeated-measures ANOVA (F-test), with an expected effect size f = 0.25 (medium effect with partial η^2^ = 0.06), α = 0.05 and power = 0.8, yielding a required sample size of 21. Therefore, our included sample size met the statistical requirements.

## 3. Results

Baseline descriptive data are presented in Table 2. The subjects were 19.8 ± 0.9 years old, with a height of 174.0 ± 4.0 cm, a body weight of 63.6 ± 5.1 kg, and 1.24 ± 0.5 years of badminton training experience. All 25 subjects completed the high-intensity field test without experiencing sports injuries or other exercise-related accidents. Post-exercise, the mean subjective fatigue rating (RPE) increased from 6 to 18.72 ± 1.31, indicating that the high-intensity field test induced peripheral fatigue levels in the subjects (Figure 2).

### 3.1. Trend of Changes in HRV Across Time Points

**Figure 3:**
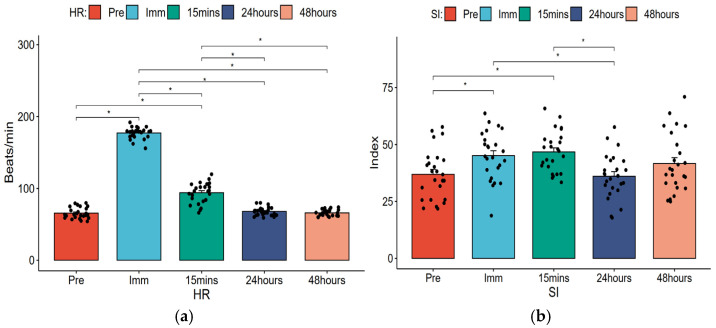
Changes in HR and SI at different time points and the distribution of samples. (**a**) HR; (**b**) SI; * *p* < 0.05, using Bonferroni adjustment.

**Figure 4:**
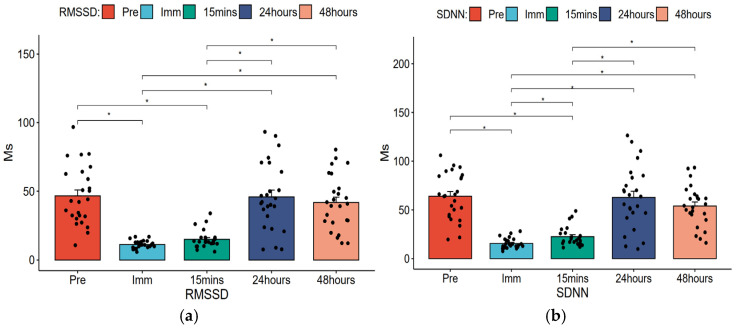
Changes in time-domain parameters of HRV at different time points and the distribution of samples. (**a**) RMSSD; (**b**) SDNN; * *p* < 0.05, using Bonferroni adjustment.

**Figure 5:**
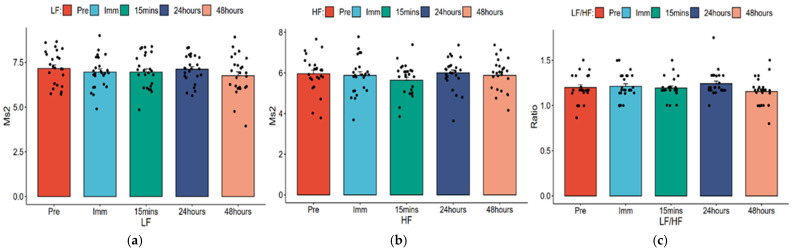
Changes in frequency-domain parameters of HRV at different time points and the distribution of samples. (**a**) LF; (**b**) HF; (**c**) LF/HF.

Repeated ANOVA was used to analyze the trend of changes in HRV across time points and post hoc tests were performed with *p* values adjusted using the Bonferroni method. Table 3 displays the mean changes in HRV across time points, and Figure 3, Figure 4 and Figure 5 present bar charts illustrating the HRV change trends and sample size distributions at each time point.

#### 3.1.1. HR and SI

Table 3 and Figure 3 show that HR increased from a baseline pre-test value of 65 beats/min to 177 beats/min immediately post-exercise and 94 beats/min at 15 min post-exercise, with statistically significant differences (*p* < 0.05). At 24 and 48 h, HR returned to pre-test levels of 68 beats/min and 66 beats/min, respectively, showing no significant differences (*p* > 0.05). Additionally, HR changes immediately, 15 min, and 24 h post-exercise were statistically significant (*p* < 0.05). The overall temporal trend was statistically significant (*p* < 0.001, partial η^2^ = 0.973, large effect size).

SI increased from a pre-test value of 36.92 to 45.16 immediately post-exercise and 46.76 at 15 min post-exercise, with statistically significant differences (*p* < 0.05). At 24 and 48 h post-exercise, values were 36.08 and 41.68 respectively, showing no significant differences (*p* > 0.05). Although no statistical significance was observed, the SI value at 48 h post-exercise increased to 41.68. SI changes between immediately post-exercise or 15 min and 24 h post-exercise were statistically significant (*p* < 0.05). The overall temporal trend was statistically significant (*p* < 0.001, partial η^2^ = 0.236, large effect size).

#### 3.1.2. Time-Domain Parameters of HRV

Table 3 and Figure 4 show that RMSSD decreased from a baseline pre-test value of 46.7 ms to 11.22 ms immediately post-exercise and to 15.09 ms at 15 min post-exercise, with statistically significant differences (*p* < 0.05). At 24 and 48 h post-exercise, RMSSD recovered to pre-test levels of 45.96 ms and 41.85 ms respectively, showing no significant differences (*p* > 0.05). Additionally, RMSSD changes between immediately or 15 min post-exercise and 24 or 48 h post-exercise were statistically significant (*p* < 0.05). The overall temporal trend was statistically significant (*p* < 0.001, partial η^2^ = 0.553, large effect size).

SDNN decreased from the pre-test value of 63.93 ms to 15.51 ms immediately post-exercise and 22.62 ms at 15 min post-exercise, with statistically significant differences (*p* < 0.05). At 24 or 48 h, values were 62.78 ms and 53.96 ms respectively, showing no significant differences (*p* > 0.05). SDNN changes at immediate, 15 min, and 24 h were statistically significant (*p* < 0.05). The overall temporal trend showed statistical significance (*p* < 0.001, partial η^2^ = 0.586, large effect size).

#### 3.1.3. Frequency-Domain Parameters of HRV

Table 3 and Figure 5 show changes in three indicators (LF, HF, and LF/HF) across five time points before and after the high-intensity field test. Neither the overall trend test nor post hoc pairwise comparisons showed statistical significance (all *p* > 0.05).

### 3.2. HRV Differences from Baseline and Effect Sizes

Table 4, Table 5 and Table 6 present further analysis results, showing HRV differences from baseline at different time points post-exercise and their effect sizes.

Table 4 shows that using pre-test HR as a reference, HR differed significantly immediately post-exercise (difference: −111.56 (95% CI: −119.27, −103.84), *p* < 0.001, Hedges’ g = 14.39, large effect size) and 15 min post-exercise (difference: −28.40 (95% CI: −37.10, −19.69), *p* < 0.001, Hedges’ g = 2.49, large effect size). Similarly, using pre-test SI as a reference, SI differed significantly immediately post-exercise (difference: −8.24 (95% CI: −15.85, −0.63), *p* < 0.001, Hedges’ g = 0.77, large effect size) and 15 min post-exercise (difference: −9.84 (95% CI: −16.61, −3.07), *p* < 0.001, Hedges’ g = 0.99, large effect size).

Table 5 shows that using pre-test RMSSD as a reference, RMSSD differed significantly immediate post-exercise (difference: 35.44 (95% CI: 21.95, 48.93), *p* < 0.001, Hedges’ g = 2.31, large effect size) and 15 min post-exercise (difference: 31.64 (95% CI: 17.85, 45.42), *p* < 0.001, Hedges’ g = 1.99, large effect size). Similarly, using pre-test SDNN as reference, immediate post-exercise (difference: 48.44 (95% CI: 32.49, 64.38), *p* < 0.001, Hedges’ g = 2.78, large effect size) and 15 min post-exercise (difference: 41.48 (95% CI: 26.23, 56.72), *p* < 0.001, Hedges’ g = 2.25, large effect size).

Table 6 shows that using pre-test as reference, differences in LF, HF, and LF/HF were not statistically significant at any time point (all *p* > 0.05).

## 4. Discussion

We interpret and discuss the findings of this study in conjunction with the previous research. The results indicate that sport-specific high-intensity interval exercise leads to significant parasympathetic withdrawal in non-professional badminton players, with recovery dynamics that differ from those reported in professional athletes. The frequency-domain indices were insensitive to these acute changes, a phenomenon that raises important considerations for optimal HRV monitoring protocols in amateur sports settings.

A single bout of intermittent badminton field testing elicited a large effect on autonomic perturbation (RMSSD decline of 63%, g = 2.14) in male non-elite players, exceeding the magnitude suggested for athlete monitoring [27]. This magnitude is comparable to recent data on recreational soccer players after two Yo-Yo IR1 bouts (RMSSD decline of 55%, d = 1.7) [25]. In addition, this study found that a single bout of intermittent high-intensity badminton field testing elicited significant changes in cardiac autonomic modulation in male non-elite athletes: immediately to 15 min post-exercise, SI significantly increased (sympathetic activation), and time-domain indicators RMSSD and SDNN markedly decreased (parasympathetic inhibition). These alterations returned to baseline levels after 24 h; however, frequency-domain parameters (LF, HF, LF/HF) showed no significant changes. This phenomenon is consistent with the “sympathetic excitation but LF decline” paradox observed by Stanley et al. [28]. Several studies have also reported that frequency-domain analysis may lack sufficient sensitivity to detect acute shifts in sympathovagal balance [12,13]. The LF/HF interpretation requires methodological caution given ongoing controversies regarding its validity as a sympathovagal balance marker [7]. The lack of significant changes in frequency-domain parameters in this study, despite clear parasympathetic withdrawal evidenced by RMSSD, reflects the known limitations of spectral analysis during non-stationary post-exercise recovery [6]. The dominant vagal withdrawal aligns with the “central command + mechanoreflex” model recently validated by Stanley et al. using pharmacological blockade [28]. Consistent with 2023 findings in trail runners [29], we observed a “sympatho-excitation yet reduced LF” paradox, where heavy intermittent exercise suppresses rather than augments LF power. Recent studies have demonstrated that immediately post-exercise, SDNN, RMSSD, HF, and LF values significantly decreased compared to pre-exercise levels [9,10]. The HRV response to exercise modalities was similar between high-intensity exercise and moderate-intensity exercise, suggesting that this similarity may be advantageous for exercise prescription and programming from an autonomic function perspective [9]. However, these previous studies were based on small sample sizes, which may introduce random measurement errors. Our present analysis, based on 25 subjects, faces similar limitations. Different HRV measurement methodologies necessitate cautious interpretation of results [16]. The inconsistent reports regarding LF, HF, and LF/HF responses following high-intensity exercise, while requiring careful physiological interpretation, present challenges for future research.

When benchmarked against Phomsoupha et al., who used a simulated match in elite players (RMSSD decline of 42%, d = 0.5) [30], our non-elite cohort exhibited both a larger relative decrement and larger effect size (RMSSD decline of 63%, g = 2.1), underscoring their slower autonomic recovery kinetics and heightened susceptibility to training load. Compared with elite athletes, our non-elite players exhibited a more pronounced decline and slower recovery. This finding supports the hypothesis proposed by Granero-Gallegos et al. that non-elite athletes have a lower rate of parasympathetic reactivation [29].

Post-session RMSSD or SDNN assessments could be integrated into smartphone apps to flag insufficient recovery [6]. Granero-Gallegos et al. recently demonstrated that HRV-guided micro-periodization reduced injury rates by 28% in collegiate athletes, a strategy readily transferable to recreational badminton [29]. HRV-based training can reduce athletes’ injury rates; thus, it is recommended to incorporate dynamic HRV monitoring into training plans, combining it with multidimensional data such as sleep quality and training logs, to dynamically adjust training loads. The magnitude and immediacy of HRV depression support the integration of post-session RMSSD or SDNN monitoring into routine training load management, offering a low-cost, non-invasive strategy to individualize recovery and prevent over-reaching in recreational badminton populations. However, our research findings indicate that HRV frequency-domain analysis is insensitive to the acute effects of high-intensity exercise. Based on this, we recommend incorporating HRV time-domain indicators and the stress index for dynamic monitoring in high-intensity training protocols.

The strengths of this study lie in the control of time series and the effect size reporting method, which provides a direct basis for comparison with elite athletes and other populations. Although the present findings support integrating the time-domain HRV (RMSSD, SDNN) and stress index into training monitoring for recreational badminton players, several methodological and physiological constraints warrant cautious interpretation. First, breathing patterns can significantly interfere with frequency-domain HRV indices [31]; although time-domain indices are less affected by respiration, uncontrolled breathing patterns may still constitute an influencing factor. Second, sleep quality is a critical but difficult-to-control recovery regulatory factor; despite requiring participants to maintain habitual sleep schedules, unmonitored sleep structure and quality may lead to inter-individual differences in HRV recovery dynamics. Third, the single-arm uncontrolled experimental design limits causal inference; our findings are also only applicable to young male amateur athletes. Fourth, participants were provided with hydration post-exercise, but the amount of fluid intake was not specifically recorded; although all participants were healthy young adults, the hydration status may also influence the results. Future multi-time point studies should incorporate assessments of respiratory rate, hydration status, and subjective sleep quality to control for the effects of these factors on post-exercise autonomic recovery.

## 5. Conclusions

Following high-intensity exercise leading to peripheral fatigue, cardiac autonomic function demonstrates a “suppression–recovery” dynamic pattern: cardiac stress levels increase significantly within 15 min post-exercise, with decreased overall HRV regulatory capacity and strong inhibition of parasympathetic activity; HRV status may return to baseline levels after 24 h. However, the frequency-domain indices of HRV showed no significant changes in response to the acute effects of high-intensity exercise.

## Figures and Tables

**Table 1:** Description and statistical measures of HRV.

Variable	Units	Description
HRV	Ms	All NN intervals in the total number divided by the height
SI		Stress index, an integrated quantitative measure developed by combining key HRV parameters from both time-domain and frequency-domain analyses, which are fitted using an algorithm grounded in a physiological model of the autonomic nervous system
Time-domain parameters		
RMSSD	ms	Root mean square of successive differences between normal RR intervals, reflecting parasympathetic activity
SDNN	ms	Standard deviation of all normal RR intervals, representing overall autonomic modulation
Frequency-domain parameters		
LF	ms^2^	Low-frequency power (0.04–0.15 Hz), associated with both sympathetic and parasympathetic influences
HF	ms^2^	High-frequency power (0.15–0.40 Hz), primarily reflective of parasympathetic activity
LF/HF		Ratio LF(ms^2^)/HF(ms^2^), widely used as an index of sympathovagal balance

**Table 2:** Baseline demographic and characteristics of subjects.

Characteristics	Subjects (n = 25)
Age (years)	19.8 ± 0.9
Weight (kg)	63.6 ± 5.1
Height (cm)	174.0 ± 4.0
BMI (kg/m^2^)	21.5 ± 1.6
Experience of playing (years)	1.24 ± 0.5
Height with arm reach (cm)	220.9 ± 6.7
Leg length (cm)	80.9 ± 3.1
Blood pressure	
Systolic blood pressure (mmHg)	121.3 ± 18.4
Diastolic blood pressure (mmHg)	59.4 ± 11.9

Noted: All data were recorded in mean ± SD.

**Table 3:** Changes in HRV at different time points after the intermittent high-intensity field test (n = 25).

HRV	Pre-Test	Immediate	15 min	24 h	48 h	*p* for Trend	Partial η^2^
HR (beats/min)	65.64 ± 7.61	177.20 ± 7.92 ^a^	94.04 ± 14.17 ^a,b^	68.04 ± 5.91 ^b,c^	65.92 ± 4.59 ^b,c^	<0.001	0.973
SI	36.92 ± 10.86	45.16 ± 10.59 ^a^	46.76 ± 8.93 ^a^	36.08 ± 9.95 ^b,c^	41.68 ± 12.87	<0.001	0.236
Time-domain parameters							
RMSSD (ms)	46.70 ± 21.47	11.22 ± 2.98 ^a^	15.09 ± 6.30 ^a^	45.96 ± 24.30 ^b,c^	41.85 ± 19.92 ^b,c^	<0.001	0.553
SDNN (ms)	63.93 ± 24.01	15.51 ± 5.19 ^a^	22.62 ± 9.76 ^a,b^	62.78 ± 32.04 ^b,c^	53.96 ± 20.65 ^b,c^	<0.001	0.586
Frequency-domain parameters							
LF (ms^2^)	7.16 ± 0.98	6.96 ±0.88	6.96 ±0.89	7.12± 0.73	6.76 ± 1.09	0.489	0.035
HF (ms^2^)	5.96 ± 0.88	5.88± 0.88	5.64 ± 0.76	6.00 ± 0.70	5.88 ± 0.70	0.421	0.039
LF/HF	1.21 ± 0.14	1.19 ± 0.12	1.24 ±0.13	1.20 ± 0.15	1.15 ± 0.15	0.242	0.055

Noted: All data were recorded as mean ± SD. HRV, heart rate variability; HR, heart rate; SI, stress index; LF, low frequency; HF, high frequency. LF/HF, low frequency–high frequency ratio; SDNN, standard deviation of all normal to normal intervals; RMSSD, the square root of the mean of the sum of the squares of differences between the adjacent normal to normal intervals. Repeated ANOVA was used to test within groups and post hoc tests were performed with *p* values adjusted using the Bonferroni method; *p* < 0.05: ^a^, pre-test vs. post-imm, post-15 min, post-24 and 48 h; ^b^, post-imm vs. post-15 min, post-24 and 48 h; ^c^, post-15 min vs. post-24 and 48 h.

**Table 4:** Differences in HR and SI at different time points compared to baseline (n = 25).

HRV	Difference (95%CI)	*p*	Hedges’ g
HR (beats/min)			
Immediate	−111.56 (−119.27, −103.84)	<0.001	14.39
15 min	−28.40 (−37.10, −19.69)	<0.001	2.49
24 h	−2.40 (−6.98, −2.18)	1.000	0.36
48 h	−0.28 (−4.53, 3.97)	1.000	0.05
SI			
Immediate	−8.24 (−15.85, −0.63)	0.027	0.77
15 min	−9.84 (−16.61, −3.07)	0.002	0.99
24 h	0.84 (−6.62, 8.30)	1.000	0.08
48 h	−4.76 (−12.41, 2.89)	0.667	0.39

Noted: *p* value has been adjusted using the Bonferroni method.

**Table 5:** Differences in time-domain parameters of HRV at different time points compared to baseline (n = 25).

HRV	Difference (95%CI)	*p*	Hedges’ g
RMSSD (ms)			
Immediate	35.44 (21.95, 48.93)	<0.001	2.31
15 min	31.64 (17.85, 45.42)	<0.001	1.99
24 h	0.72 (−17.72, 19.16)	1.000	0.03
48 h	4.88 (−12.54, 22.30)	1.000	0.23
SDNN (ms)			
Immediate	48.44 (32.49, 64.38)	<0.001	2.78
15 min	41.48 (26.23, 56.72)	<0.001	2.25
24 h	1.28 (−24.01, 26.57)	1.000	0.04
48 h	10.00 (−8.17, 28.17)	1.000	0.44

Noted: *p* value has been adjusted using the Bonferroni method.

**Table 6:** Differences in frequency-domain parameters of HRV at different time points compared to baseline (n = 25).

HRV	Difference (95%CI)	*p*	Hedges’ g
LF (ms^2^)			
Immediate	0.20 (−0.59, 0.99)	1.000	0.21
15 min	0.20 (−0.39, 0.79)	1.000	0.21
24 h	0.04 (−0.68, 0.76)	1.000	0.05
48 h	0.40 (−0.37, 1.17)	1.000	0.38
HF (ms^2^)			
Immediate	0.08 (−0.56, 0.72)	1.000	0.09
15 min	0.32 (−0.20, 0.84)	0.727	0.38
24 h	−0.04 (−0.67, 0.59)	1.000	0.05
48 h	0.08 (−0.53, 0.69)	1.000	0.10
LF/HF			
Immediate	0.02 (−0.11, 0.15)	1.000	0.15
15 min	−0.03 (−0.14, 0.08)	1.000	0.22
24 h	0.01 (−0.10, 0.13)	1.000	0.07
48 h	0.06 (−0.06, 0.18)	1.000	0.41

Noted: *p* value has been adjusted using the Bonferroni method.

## Data Availability

Data supporting the findings of this research are available from the corresponding author because the data are not publicly available due to privacy and ethical restrictions.

## Abbreviations

The following abbreviations are used in this manuscript:

HRV	Heart rate variability
SI	Stress index
RMSSD	Root mean square of successive differences between normal heartbeats
SDNN	Standard deviation of all normal heartbeat intervals
HF	High-frequency
LF	Low-frequency
FT	High-intensity intermittent court test
HR	Heart rate
